# Clinical Relevance of VPAC1 Receptor Expression in Early Arthritis: Association with IL-6 and Disease Activity

**DOI:** 10.1371/journal.pone.0149141

**Published:** 2016-02-16

**Authors:** Iria V. Seoane, Ana M. Ortiz, Lorena Piris, Amalia Lamana, Yasmina Juarranz, Rosario García-Vicuña, Isidoro González-Álvaro, Rosa P. Gomariz, Carmen Martínez

**Affiliations:** 1 Departamento de Biología Celular, Facultad de Biología, Universidad Complutense de Madrid, Madrid, Spain; 2 Servicio de Reumatología, Hospital Universitario de la Princesa, Instituto de Investigación Sanitaria la Princesa, Madrid, Spain; 3 Unidad de Apoyo Metodológico, Hospital Universitario de la Princesa, Instituto de Investigación Sanitaria la Princesa, Madrid, Spain; 4 Departamento de Biología Celular, Facultad de Medicina, Universidad Complutense de Madrid, Madrid, Spain; University of Leuven, Rega Institute, BELGIUM

## Abstract

**Background:**

The vasoactive intestinal peptide (VIP) receptors VPAC1 and VPAC2 mediate anti-inflammatory and immunoregulatory responses in rheumatoid arthritis (RA). Data on the expression of these receptors could complement clinical assessment in the management of RA. Our goal was to investigate the correlation between expression of both receptors and the 28-Joint Disease Activity Score (DAS28) in peripheral blood mononuclear cells (PBMCs) from patients with early arthritis (EA). We also measured expression of IL-6 to evaluate the association between VIP receptors and systemic inflammation.

**Methods:**

We analyzed 250 blood samples collected at any of the 5 scheduled follow-up visits from 125 patients enrolled in the Princesa Early Arthritis Register Longitudinal study. Samples from 22 healthy donors were also analyzed. Sociodemographic, clinical, and therapeutic data were systematically recorded. mRNA expression levels were determined using real-time PCR. Then, longitudinal multivariate analyses were performed.

**Results:**

PBMCs from EA patients showed significantly higher expression of VPAC2 receptors at baseline compared to healthy donors (p<0.001). With time, however, VPAC2 expression tended to be significantly lower while VPAC1 receptor expression increased in correlation with a reduction in DAS28 index. Our results reveal that more severe inflammation, based on high levels of IL-6, is associated with lower expression of VPAC1 (p<0.001) and conversely with increased expression of VPAC2 (p<0.001). A major finding of this study is that expression of VPAC1 is lower in patients with increased disease activity (p = 0.001), thus making it possible to differentiate between patients with various degrees of clinical disease activity.

**Conclusion:**

Patients with more severe inflammation and higher disease activity show lower levels of VPAC1 expression, which is associated with patient-reported impairment. Therefore, VPAC1 is a biological marker in EA.

## Introduction

Rheumatoid arthritis (RA) is a common autoimmune disease with a heterogeneous clinical course and various pathogenic mechanisms leading to chronic synovitis and joint destruction. Macrophages, lymphocytes and other effector cell types, along with a wide range of inflammatory and growth factors, are involved in a complex network whose balance is shifted both systemically and locally towards a proinflammatory state [1]. The main features of RA are joint pain, swelling and stiffness, as well as increased levels of acute phase reactants. These features correlate with joint damage and are commonly used to assess the activity of RA [2]. Frequent measurement of disease activity is recommended by the American College of Rheumatology/European League Against Rheumatism [3], the European League Against Rheumatism [4] and the treat-to-target international task force [5], with a clear goal, namely, reducing, if not eliminating, inflammation to facilitate remission in the fastest possible way [6]. For this reason, many investigators aim to identify biological markers reflecting underlying pathophysiological processes associated with the clinical activity of RA.

Vasoactive intestinal peptide (VIP) is a broadly distributed peptide produced by neural, endocrine and immune cells. Multiple studies have shown its involvement in the maintenance of homeostasis and health [7–9].

The potent immunomodulatory functions of VIP have been demonstrated both *in vivo*, in a murine model of collagen-induced arthritis [10], and *ex vivo*, in cultured fibroblast-like synoviocytes and in peripheral blood lymphocytes of patients with rheumatoid arthritis [10, 11]. We recently reported promising findings about the role of endogenous VIP in early arthritis (EA), showing that patients with low baseline VIP levels have worse disease outcome [12]. The protective effect of VIP has been attributed to 3 key features: anti-inflammatory properties, regulation of CD4+ T-cell subpopulations and modulation of mediators involved in the destruction of bone and cartilage [10, 13–15].

VIP triggers its biological responses by interacting with 2 subtypes of the class B family of G protein–coupled receptors, VPAC1 and VPAC2 [16]. VPAC1 is constitutively expressed in monocytes/macrophages and lymphocytes [17]. VPAC2 has also been described in lymphocytes and macrophages as an inducible receptor after immune stimulation [17]. Dynamic regulation of VPAC1 and VPAC2 receptors has been reported in both physiological and pathological processes [18]. Thus, altered expression of VPAC2 receptor has been reported in monocytes isolated from patients with Sjögren’s syndrome [19] and in activated CD4+ T cells from patients with multiple sclerosis [20]. Furthermore, decreased VPAC1 surface expression in peripheral blood mononuclear cells (PBMCs) [21] and increased VPAC2 expression in fibroblast-like synoviocytes [22] have been demonstrated in RA. Although these findings reveal abnormal expression of VIP receptors in autoimmune diseases, their association with clinical course has not been investigated.

Given the ability of VIP to regulate the intensity of the inflammatory process and the immune response, together with the particular expression pattern of VIP receptors in RA, we hypothesized that expression of VPAC1 and VPAC2 might be associated with disease activity and would thus reflect the patient’s clinical status.

Therefore, our goal was to investigate the association between the expression of VIP receptors during the follow-up of patients with EA and the degree of systemic inflammatory activation. To do so, we evaluated IL-6 expression levels and assessed the well-recognized disease activity score based on the assessment of 28 joints (DAS28) [23].

## Materials and Methods

### Ethics statement

This study was undertaken in compliance with the Declaration of Helsinki. The protocol for the Princesa Early Arthritis Register Longitudinal (PEARL) study was reviewed and approved by the Ethics Committee for Clinical Research at the Instituto de Investigación Sanitaria La Princesa. All patients were informed about the study and signed an informed consent form before inclusion in the register.

### Patients and Controls

The PEARL study includes patients with more than 1 swollen joint for at least 4 weeks and symptoms for less than a year. The register protocol includes collection of socio-demographic, clinical and therapeutic data, as well as samples at each of the 5 scheduled visits (baseline, 6, 12, 24 and 60 months). A more detailed description of the protocol has been published elsewhere [12].

For the present study, we used 250 blood samples collected during the follow-up of 125 patients enrolled in the PEARL study. Among these, 79 fulfilled the 2010 American College of Rheumatology/European League Against Rheumatism criteria for Rheumatoid Arthritis [24] and 46 patients were diagnosed with chronic undifferentiated arthritis. None of these 46 patients fulfilled the criteria for other inflammatory disorders (infectious arthritis, microcrystalline arthritis, connective tissue diseases, spondyloarthritis or psoriatic arthritis). Regarding received treatment, there were 85 visits in which patients were not under disease modifying antirheumatic drugs (DMARD) treatment, mostly baseline visits and few follow-up visits from patients with mild disease. In 128 visits patients were under DMARD monotherapy: 65% on methotrexate (MTX), 20% on leflunomide (LEF), 10% on antimalarials (AM) and other DMARDs 5%. Finally, in 37 visits patients received combined DMARD treatment with a very heterogeneous profile including MTX+LEF (40%), MTX+AM (18%) and TNF-blockers+different DMARDS (26%), other combinations included MTX+SSZ, LEF+SSZ, LEF+AM and so on.

Healthy donors (n = 22) were recruited from Hospital Universitario de La Princesa. This group included 9 women and 13 men, whose median and interquartile range (IQR) age was 38 (30–49).

### Semiquantitative real-time reverse transcription-polymerase chain reaction (RT-PCR) assay

PBMCs from EA patients and blood samples from healthy donors were isolated using Ficoll density gradient centrifugation (Histopaque-1077™, Sigma-Aldrich). The expression of VPAC1, VPAC2 and IL-6 in PBMCs was analyzed by RT-PCR. Total RNA was obtained from PBMCs using TRI Reagent™ (Sigma-Aldrich), and 2 μg was reverse transcribed using the High Capacity cDNA Reverse Transcription Kit (Applied Biosystems). Complementary DNA (25 ng/well) was amplified by semiquantitative real-time PCR performed with TaqMan Gene Expression Master Mix (Applied Biosystems) using manufacturer-predesigned primers (Applied Biosystems. VPAC1: Hs00270351_m1; VPAC2: Hs00173643_m1; IL-6: Hs00985639_m1). Data were normalized for relative quantification and expressed in reference to the β-actin housekeeping gene using the 2^-ΔΔCt^ formula, as previously described [22].

### Statistical analysis

Descriptive analysis: quantitative variables were represented as the mean ± standard deviation (SD). Variables with equal variances were analyzed using the *t* test or ANOVA (analysis of variance). Comparisons between the groups were made a posteriori using multiple comparison tests. The Mann Whitney or Kruskal-Wallis test was used for variables with unequal variances, and intergroup comparison was done by the multiple comparison test—non-parametric posteriori test. Homoscedasticity was assessed using Levene’s test and an ANOVA or *t*- test (n>30). Normality was not assessed. The trend test was used to study the evolution of VPAC1, VPAC2, the VPAC1:VPAC2 ratio and IL-6 during follow-up.

The association between expression of VPAC receptors and disease activity based on DAS28 was analyzed using univariate and multivariate GEE (generalized estimating equations method) with gaussian family and identity link function. Variability in the statistics derived from visit observations was analyzed using exchangeable correlation structures. The multivariate model included variables with p<0.20 in the univariate analysis. Considering that the raw data for gene expression contained left-shifted values, we decided to censor values above the 95th percentile. An appropriate transformation was applied to perform the analysis in cases of non-normal distribution. The association between quantitative variables was analyzed using Spearman’s correlation test. p values ≤0.05 were considered statistically significant. The statistical analyses were performed using Stata 13 for Windows (StataCorp LP, College Station, Texas, USA).

## Results

### Patient characteristics. Expression of VPAC1 and VPAC2 in PBMCs during follow-up

A total of 125 patients with RA (n = 79) or chronic undifferentiated arthritis (n = 46) were included in the study. Patients who fulfilled the 1987 criteria for RA [25] had more severe disease at baseline and a higher positivity of 2 validated biomarkers of diagnosis and severity (rheumatoid factor [RF] and anti-citrullinated peptide antibodies [ACPA]) than patients with undifferentiated arthritis (Table 1).

**Table 1:** Baseline characteristics of patients with early arthritis.

	Rheumatoid Arthritis (n = 79)	Undifferentiated Arthritis (n = 46)	Total (n = 125)	p-value
Age* (years)	53 [45–70]	52 [40–68]	52 [44–68]	NS
Gender** (female)	82	76	80	NS
Ethnicity** (caucasian)	64 (81%)	41 (89.1%)	105 (84%)	NS
Disease duration* (months)	5.4 [3.1–8.1]	4.6 [2.2–7]	5.1 [2.8–7.7]	0.1
GDA Pat*	50 [30–64]	42 [23–50]	47 [27–58]	0.003
GDA Phy*	50 [32–65]	26 [10–47]	41 [23–55]	<0.001
HAQ*	1.125 [0.750–1.750]	0.875 [0.375–1.125]	1 [0.625–1.500]	0.002
CRP (mg/dl) *	0.9 [0.3–2.3]	0.4 [0.2–0.9]	0.7 [0.2–1.7]	0.014
ESR (mm/h)*	32 [20–55]	21 [13–37]	27 [16–50]	0.036
DAS28 (0–10)*	5.1 [3.7–6.1]	3.6 [2.6–4.8]	4.5 [3.4–5.6]	<0.001
RF+**	72	11	50	<0.001
ACPA+**	66	11	46	<0.001

Data are shown as the *median [interquartile range] or, **n (%). GDA: Global Disease Assessment; Pat: patient; Phy: physician; HAQ: health assessment questionnaire; CRP: C-reactive protein; ESR: erythrocyte sedimentation rate; DAS28: 28-joint disease activity score; RF: rheumatoid factor; ACPA: anti-citrullinated peptide antibodies. Statistical significance was established using the Pearson chi-square test for a p value˂0.05. NS: non-significant.

**Fig 1:**
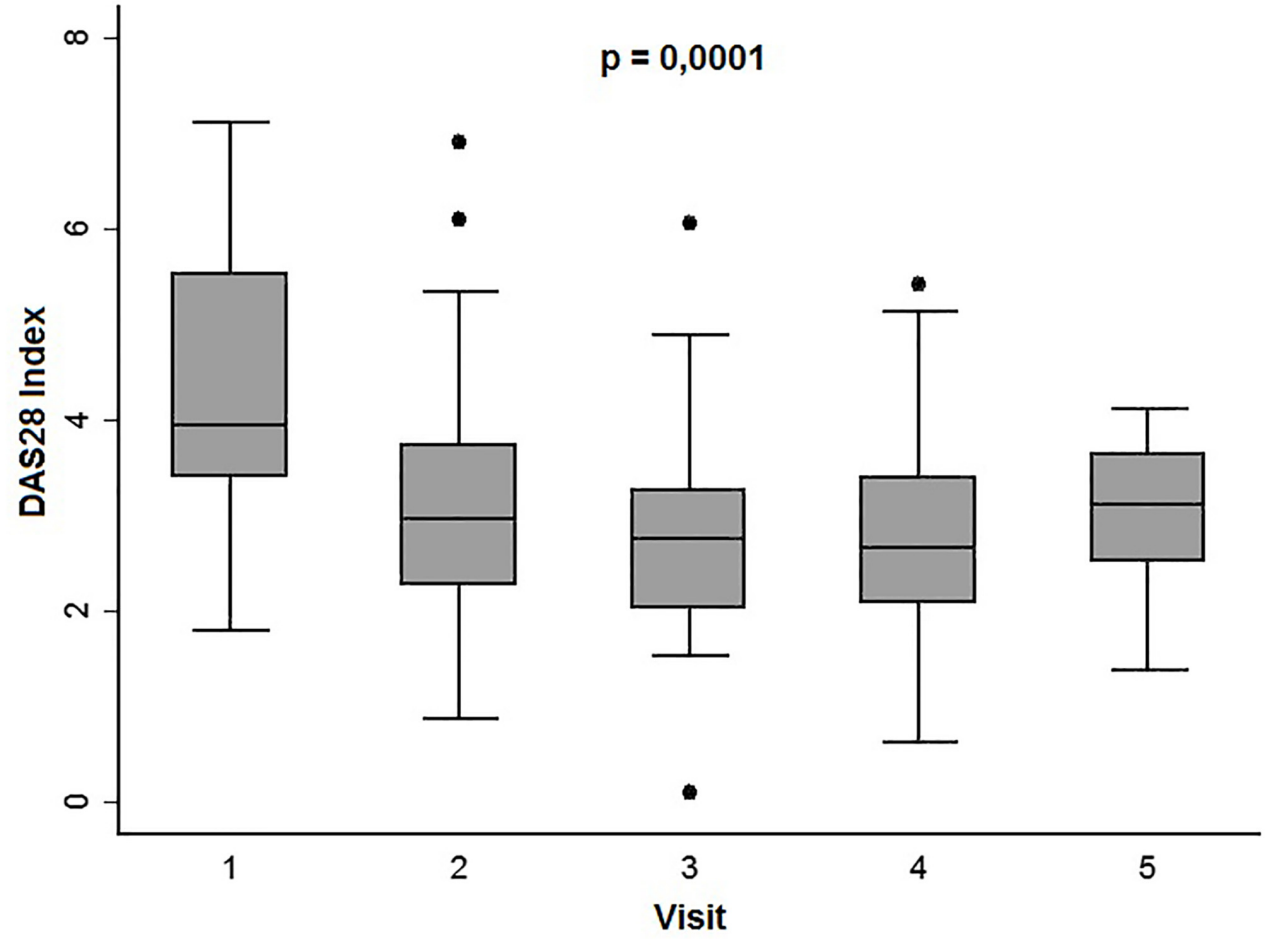
Progress of disease activity estimated by DAS28 at follow-up visits. Data are presented as the interquartile range (p75 upper edge of the box, p25 lower edge, p50 midline), p90 (line above the box), and p10 (line below the box). Dots represent outliers. Statistical significance was established using the Kruskal-Wallis test.

During follow-up protocol, disease activity was evaluated. As shown in Fig 1, significantly lower scores in disease activity were observed over the course of the disease as a consequence of adjusting treatment with glucocorticoids or DMARDs (see Materials and Methods).

We first analyzed the gene expression of both subtypes of VIP receptors (VPAC1 and VPAC2) in PBMCs isolated at baseline visit from EA patients and from controls. Slightly lower expression of VPAC1 was found in EA patients, although the difference was not statistically significant (1.2 ± 0.1 *vs* 1.6 ± 0.2, respectively; p = 0.1, Mann-Whitney test). In contrast, PBMCs from patients exhibited significantly higher mRNA levels of VPAC2 than PBMCs from healthy donors (1.7 ± 0.3 *vs* 0.8 ± 0.1, respectively; p<0.001, Mann-Whitney test).

**Fig 2:**
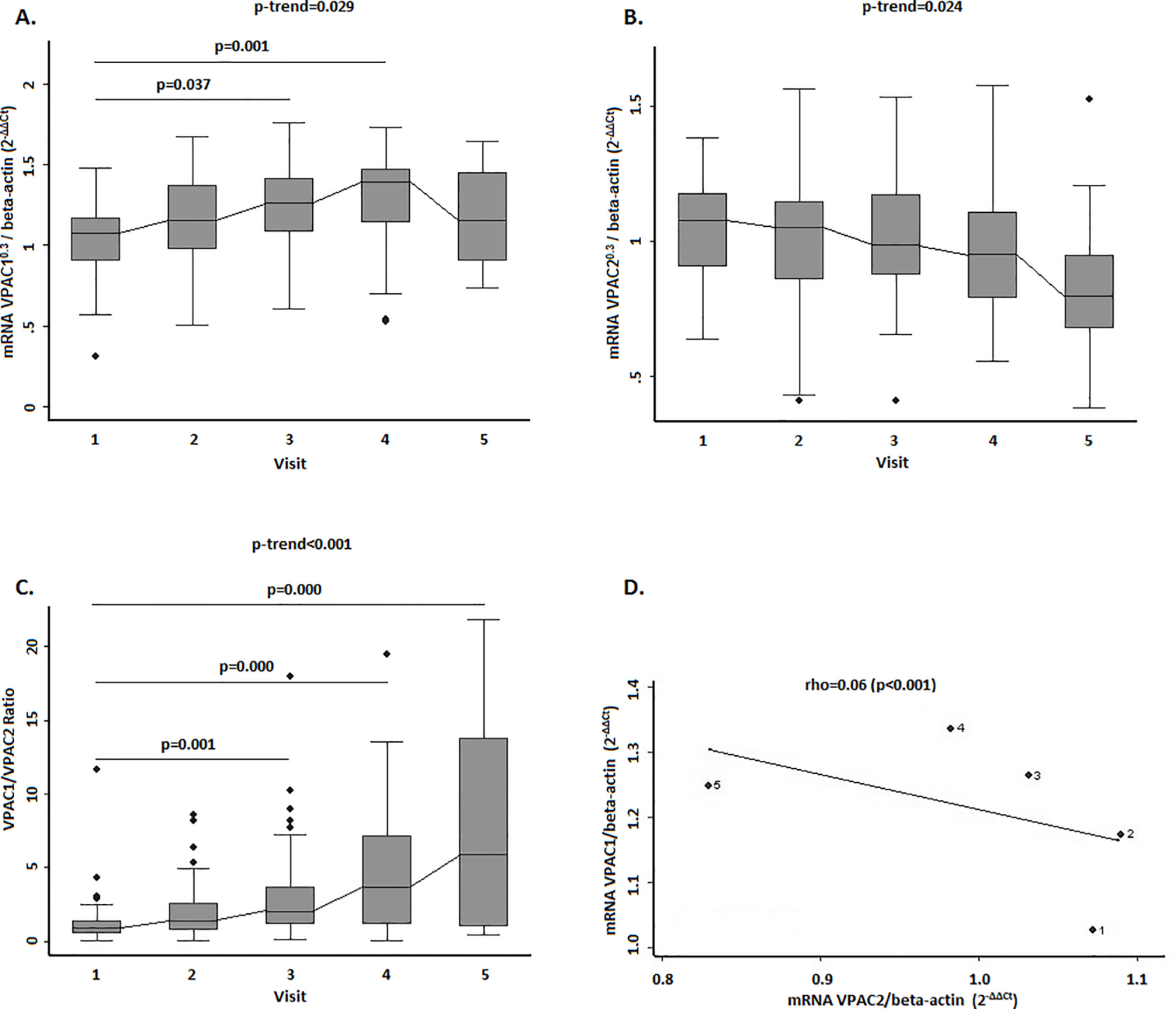
Evolution of gene expression levels of VPAC1 and VPAC2 in PBMCs from EA patients. **(A) and** (**B)** Variation in gene expression levels of VPAC1 (panel A) and VPAC2 (panel B) receptors at follow-up visits. **(C)** Evolution of the ratio of gene expression of both receptors during the follow-up. **(D)** Correlation between gene expression levels of VPAC1 and VPAC2 receptors. Statistical significance was established using the Trend test and non-parametric posteriori test (Dunnett) for intergroup comparison, in panels A, B and C. In panel D, the significance level was obtained by means of a generalized linear model nested by patient and visit. In panel D dots represent average data from patients clustered by visits from 1 to 5. Data of A, B and C panels are represented as the interquartile range (p75 upper edge of the box, p25 lower edge, p50 midline), p90 (line above the box), and p10 (line below the box) of the expression of VPACs receptors. Mathematical transformation for VPAC expression: raised to 0.3. Dots represent outliers.

Next, we studied expression of VPAC1 and VPAC2 during follow-up. As Fig 2A shows, expression of VPAC1 increased significantly (p-trend = 0.029), reaching the highest values after 2 years of follow-up. Conversely, VPAC2 expression decreased (p-trend = 0.024, Fig 2B). (Ct values of real-time PCR assay are shown in S1 Table). In order to assess the relationship between both receptors during follow-up, we evaluated the VPAC1:VPAC2 ratio in PBMCs for each patient. The ratio had increased during follow-up (from 1.3 ± 0.3 at baseline to 7.7 ± 1.6 at 5 years), suggesting dynamic regulation of both receptors (Fig 2C). After adjustment for visits, an inverse correlation between both receptors was confirmed (r = –0.60, p<0.001); therefore, increased expression of VPAC1 is associated with decreased expression of VPAC2 (Fig 2D).

As a consequence of limitations in systematic RNA extraction at the beginning of the register, many patients lacked data for all 5 visits. We therefore performed a new analysis considering only those patients with samples from 3 or 4 visits including baseline and similar results were obtained (S1 Fig).

### Correlation between VPAC receptors and IL-6 expression in EA patients

**Fig 3:**
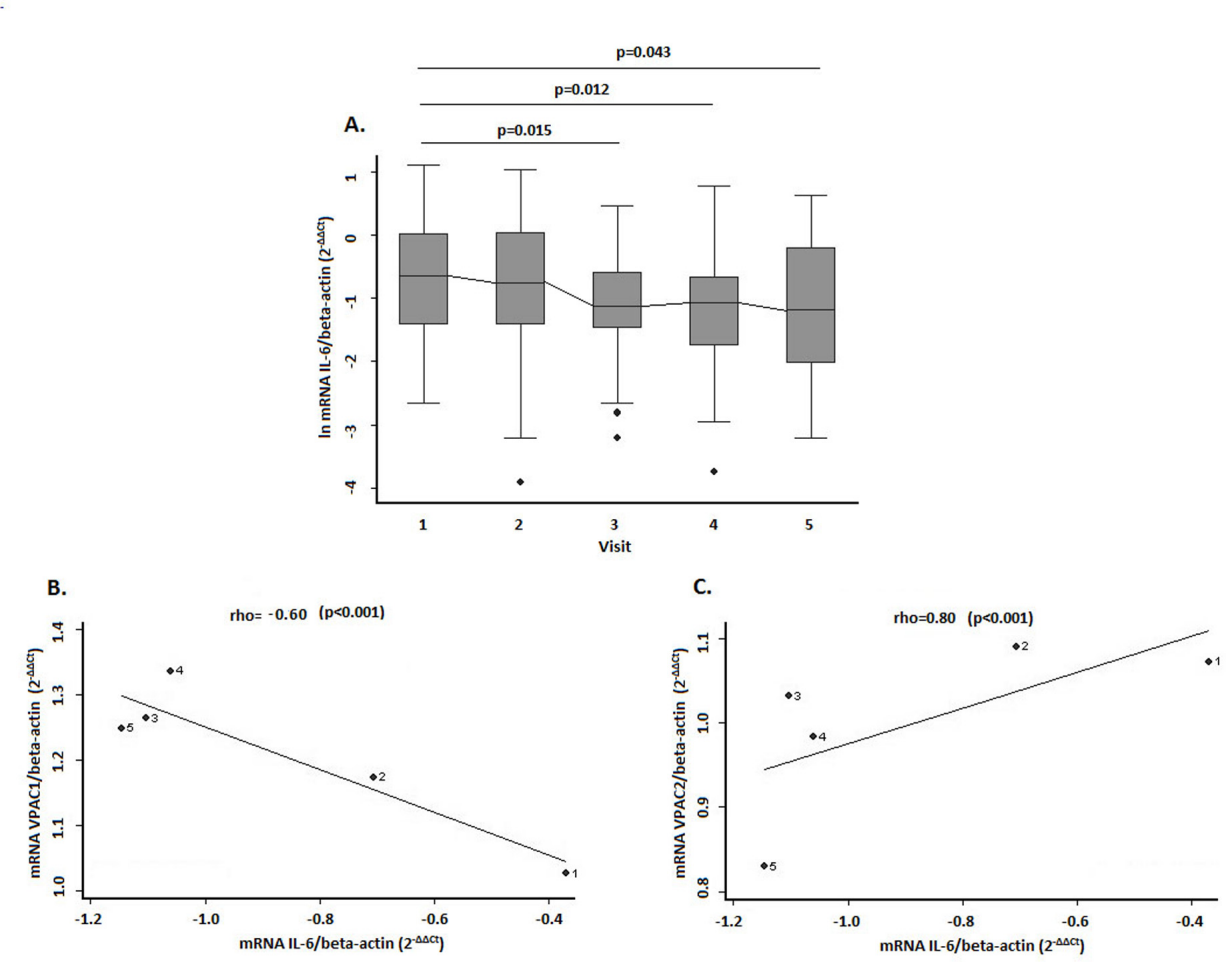
Correlation between gene expression levels of VPACs receptors and IL-6 in PBMCs from EA patients. **(A)** Evolution of IL-6 expression in the EA cohort during the follow-up. Data are represented as the interquartile range (p75 upper edge of the box, p25 lower edge, p50 midline), p90 (line above the box), and p10 (line below the box) of the expression of IL-6. Dots represent outliers. **(B) and (C)** Correlation between gene expression of VPAC1 and IL-6 (panel B) and between VPAC2 and IL-6 (panel C). Statistical significance was established using the Trend test and non-parametric posteriori test (Dunnett) for intergroup comparison, in panel A. In panels B and C the significance level was obtained by means of a generalized linear model nested by patient and visit. In panels B and C dots represent data from patients clustered by visits from 1 to 5.

IL-6 is considered a marker of systemic inflammation in RA [26]. Therefore, we decided to investigate whether there was an association between IL-6 levels and expression of VIP receptors. First, we evaluated the evolution of IL-6 expression in our EA cohort. As expected, IL-6 levels were highest at the beginning of follow-up and decreased over time (Fig 3A). (Ct values of real-time PCR assay was shown in S1 Table). We then performed a correlation analysis adjusted for the visits. As shown in Fig 3B, expression of VPAC1 was significantly lower at visits where a higher level of IL-6 was detected. Conversely, a significant and positive correlation was detected between this inflammatory marker and VPAC2 expression (Fig 3C).

### Association between higher DAS28 levels and reduced VPAC1 receptor expression. Evaluation of subgroups by DAS28 level

Given that the most obvious change during follow-up is that patients improved once they are treated [27], we next analyzed the expression of the receptors taking into account the level of disease activity (remission, low, moderate or high) assessed through DAS28. We performed a multivariate analysis to ensure a more accurate appraisal. Our data showed that higher levels of DAS28 were associated with lower gene expression of VPAC1 (Table 2). Similar results were also observed at protein level (S2 Fig). In addition, VPAC1 expression tended to be significantly higher in patients treated with combinations of disease-modifying antirheumatic drugs and in Latin American patients. We did not detect any association between VPAC2 expression and the variables studied (Table 3).

**Table 2:** Multivariate analysis of variables associated with VPAC1 expression during follow-up of patients with early arthritis.

		β Coeff.	95% CI	p-value
**DAS28**		-0.070	[-0.10–-0.300]	0.000
**Corticosteroids**		-0.002	[-0.10–0.004]	0.483
**DMARDs**				
	***None***	Ref.		
	***Monotherapy***	0.080	[-0.003–0.170]	0.059
	***Combined therapy***	0.220	[0.090–0.340]	0.001
**Origin**				
	***Spain***	Ref.		
	***South America***	0.140	[0.020–0.270]	0.025
	***Eastern Europe***	0.290	[-0.270–0.850]	0.304

The longitudinal multivariate analysis was performed with data (mathematical transformation for VPAC1 expression: raised to 0.3) from 207 visits corresponding to the 79 rheumatoid arthritis patients with all information available for ≥2 visits. β Coeff.: β coefficient of the Wald test; CI: confidence interval; DAS28: 28-joint disease activity score; DMARD: disease-modifying antirheumatic drugs. The table shows all the variables reaching p˂0.2 in the bivariate analysis.

**Table 3:** Multivariate analysis of variables associated with VPAC2 expression during follow-up of patients with early arthritis.

		β Coeff.	95% CI	p-value
**DAS28**		-0.01	[-0.04–0.02]	0.540
**Corticosteroids**		-0.0002	[-0.01–0.01]	0.940
**DMARDs**				
	***None***	Ref.		
	***Monotherapy***	0.003	[-0.08–0.08]	0.946
	***Combined therapy***	0.05	[-0.06–0.17]	0.367
**Origin**				
	***Spain***	Ref.		
	***South America***	0.001	[-0.11–0.12]	0.992
	***Eastern Europe***	0.45	[-0.07–0.98]	0.090

The longitudinal multivariate analysis was performed with data from 210 visits corresponding to the 79 rheumatoid arthritis patients with all information available for ≥2 visits. β Coeff.: β coefficient of the Wald test; CI: confidence interval; DAS28: 28-joint disease activity score; DMARD: disease-modifying antirheumatic drugs. The table shows all the variables reaching p˂0.2 in the bivariate analysis.

**Fig 4:**
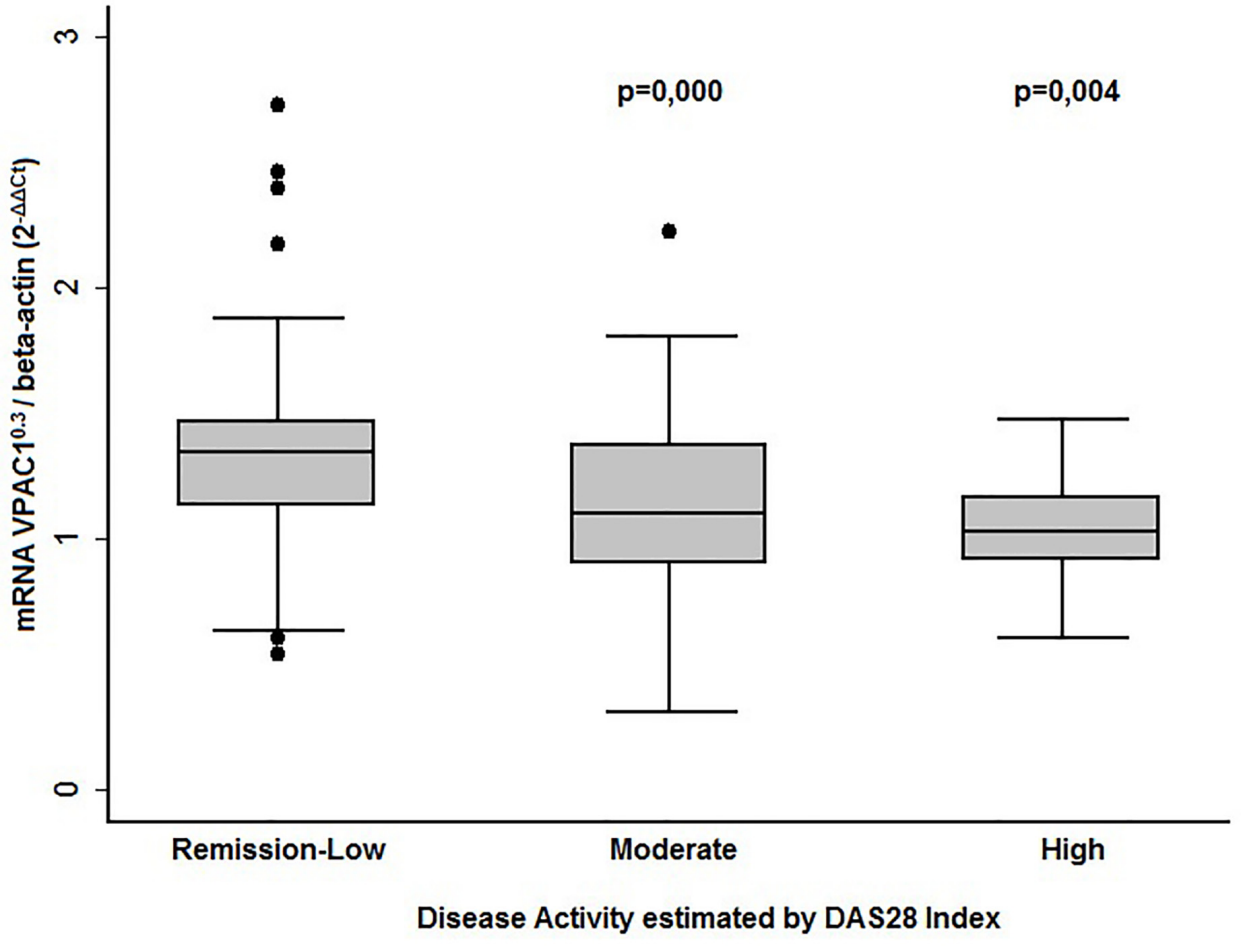
Correlation between VPAC1 gene expression levels and disease activity. Data are represented as the interquartile range (p75 upper edge of the box, p25 lower edge, p50 midline), p90 (line above the box) and p10 (line below the box). Mathematical transformation for VPAC1 expression: raised to 0.3. Dots represent outliers. Statistical significance was established using the Kruskal-Wallis test.

We analyzed VPAC1 expression in EA patients clustered into 3 sub-groups by their DAS28 levels (remission-low, moderate and high disease activity). As Fig 4 shows, patients with moderate or high activity expressed significantly lower levels of VPAC1. By contrast, patients in remission displayed higher VPAC1 expression.

### Correlation between VPAC1 expression and global disease assessed by patients and physicians

Patient perspectives have become an increasingly relevant component of RA outcome measurement, and the physician’s global assessment is the only way to quantify the patient’s opinion of disease course [28]. Therefore, patient and physician global scores were analyzed. A negative correlation was observed between VPAC1 expression and global disease activity both by the patient and by the physician (Table 4). As VPAC1 seems to be a marker of disease activity in EA, we decided to compare it with 2 acute phase reactants that are commonly used to monitor disease activity in RA, namely, the erythrocyte sedimentation rate (ESR) and C-reactive protein (CRP). Both of them correlate well with IL-6 levels, however it has been described that they are subject to a gender bias [29]. Consequently, a more useful biological marker for disease activity is needed.

**Table 4:** Correlation between VPAC1 expression and global disease assessment.

	ESR		CRP		VPAC1	
	β Coeff.	p-value	β Coeff.	p-value	β Coeff.	p-value
**GDAPa**	0.182	0<0.001	0.331	<0.001	-0.219	0.002
**GDAPh**	0.268	<0.001	0.468	<0.001	-0.305	<0.001

Spearman’s correlation test performed with quantitative variables GDAPa: global disease activity assessed by patient; GDAPh: global disease activity assessed by physician. β Coeff.: β coefficient of Spearman’s test. Statistical significance was set at p˂0.05.

Since ESR is included in the DAS28, we used global disease assessment by the physician and the patient as an independent gold standard to compare VPAC1 expression with ESR and CRP values. As shown in Table 4, although serum CRP correlated slightly better with both assessments than VPAC1 expression, VPAC1 was more useful than ESR in estimating global disease activity (Table 4).

## Discussion

Knowledge of deregulated key factors in the pathophysiological processes of RA can complement clinical assessment [30]. Many studies have emphasized the importance of VIP and their receptors in modulating inflammatory and immune responses in RA [10, 11, 13, 31]. In recent years, VIP levels have emerged as a suitable prognostic biomarker in EA, since patients who are unable to up-regulate VIP have a poorer clinical course [12]. In the present study, we provide new evidence of the functional relevance of this peptide through the association between the level of expression of VPAC1 in PBMCs and the patient’s clinical status.

VPAC1 is expressed at slightly lower levels in the PBMCs of patients with EA than in those of healthy donors, although the difference is not significant. VPAC2, however, is expressed at higher levels in patients with EA. Using flow cytometry, Delgado et al [21] showed decreased expression of VPAC1 in PBMCs from patients with RA, probably as a result of altered gene regulation, although no data were reported for RNA expression [21]. The increased expression of VPAC2 receptors shown here might reflect a compensating mechanism operating *in vivo*. In fact, the present study is the first to demonstrate a converse change in the expression of VPAC1 and VPAC2 in PBMCs during the course of EA.

Previous studies demonstrated polarization toward a Th1/Th17 response and exacerbated experimental autoimmune encephalomyelitis in mice lacking the immune-inducible VPAC2 receptor [32, 33]. In addition, transgenic mice expressing VPAC2 display a shift in CD4 T-cell polarization towards a Th2 phenotype [33, 34]. Our findings indicate that lower expression of VPAC1 in PBMCs is correlated with an increase in VPAC2 expression that could be explained as an attempt to counteract the imbalance of Th1/17 and Th2 in RA. Indeed, in Th17-polarized cells from EA patients, expression of VPAC2 predominates over VPAC1 [35]. Moreover, VPAC2 is able to mediate anti-inflammatory effects when expression of VPAC1 is reduced [22]. Future studies will examine the functional relevance of the higher expression of VPAC2 receptors in the PBMCs of patients with EA and whether this constitutes a compensatory mechanism or is simply an event brought about by damage associated with chronic inflammation.

The sustained high expression of IL-6 is a key inducer of systemic inflammation in RA and is associated with disease activity [36]. In our cohort, expression of IL-6 decreased during follow-up, probably as a result of the effectiveness of disease-modifying antirheumatic drugs. Our results reveal that more severe inflammation, seen as high levels of IL-6, is associated with lower expression of VPAC1 and, conversely, with increased expression of VPAC2. Interestingly, a major finding of the present study is that patients with a higher degree of disease activity show lower expression of VPAC1. In addition, expression of VPAC1 makes it possible to differentiate between patients with various degrees of clinical disease activity. VIP signaling through VPAC1 considerably inhibits the G1/S transition of the cell cycle in human T cells, and the expression of this receptor is down-regulated in activated T-cells [17, 18]. Thus, VPAC1 signaling appears to block the signal that causes its down-regulation and might be the reason why low expression of VPAC1 has been observed in cell lines and blasts from patients with leukemia [18]. Given that proliferative response and hyperactivity of CD4+ T cells are characteristics that contribute to the pathogenesis of RA, expression of VPAC1 could serve as an indicator of deregulated activation of the immune system. To date, we do not know whether changes in VPAC1 expression are a cause or a consequence of the disease, although our results suggest a protective role for this receptor in EA. The multiplicity and diversity of activities exerted when VPAC1 binds to VIP consistently supports this possibility. In fact, both animal and human models of RA have demonstrated that selective activation of VPAC1 is more effective for controlling the immune response than VPAC2 agonists [37]. Accordingly, treatment with a VPAC1 agonist, but not with a VPAC2 agonist, reduced the frequency of arthritis, ameliorated symptoms, and prevented joint damage in an experimental model of arthritis [7]. In addition, treatment with a VPAC1 agonist decreased the inflammatory response during progression of collagen-induced arthritis by down-regulating the production of various inflammatory mediators and reduced the presence of autoreactive Th1 cells in the joints [21].

The fact that patients treated with monotherapy or combinated therapy exhibit higher levels of VPAC1 expression but not VPAC2 suggests that VPAC1 receptor regulation is probably sensitive to disease-modifying antirheumatic drugs, as shown for several cytokines and their receptors [38, 39]. Antirheumatic agents could act indirectly by modifying the profile of immune cell types bearing VPAC1/2 receptors within the population of PBMCs. In fact, combined therapy increased the number of lymphocytes and monocytes (S3 Fig) which was associated with increased expression of VPAC1 but it was not correlated with VPAC2 expression (S2 Table). However, the lymphocytes:monocytes ratio was not affected by prescribed treatments and no association was observed with VPACs receptors expression (S3 Fig). Moreover, anti-inflammatory agents could change the relative proportions of specific lymphocyte subpopulations expressing VPACs receptors. In this sense, it has been described that Th17 polarization induces a higher expression of both receptors in EA patients mainly of the VPAC2 receptor [35]. Thus, a shift in the relative levels of the different T cell subpopulations during treatment could eventually result in changes in the VPAC1 expression. Finally, DMARD might act directly by regulating the expression of VPAC1 receptor on lymphocytes and monocytes.

Therefore, VPAC1 could serve as an indicator for clinical observation of disease activity and also as a therapeutic target in RA.

Finally, our results reveal that expression of VPAC1 correlates with global disease activity as estimated by patients and physicians in much the same way as currently used biomarkers of disease activity. This observation is interesting, because the patient’s perception has been repeatedly shown to have crucial predictive value [40]. Although patient perspectives *per se* are subjective, they will likely serve as a benchmark to improve not only traditional objective measures of disease activity, but also patient satisfaction [41].

The mechanisms involved in the regulation of the expression of VPAC1 in immune cells from patients with EA remain unknown. Although predisposition to RA has been associated with a haplotype in the VPAC1 3´-UTR [21], no evidence of an association with clinical variables has emerged. Ongoing and future studies investigating the molecular mechanisms regulating VPAC1 are essential for better understanding of its role in inflammatory disease and autoimmunity.

In conclusion, patients with more severe inflammation and high disease activity show lower levels of VPAC1 expression associated with patient-reported impairment. Although the mechanisms of VPAC1 regulation have yet to be elucidated, emerging evidence supports the clinical relevance of VPAC1 expression in RA.

## Supporting Information

S1 Appendix Materials and Methods of Western blot analysis of VPAC1 receptor. (DOC)

S1 Fig Evolution of gene expression levels of VPAC1 and VPAC2 in PBMCs from EA patients with 3 or 4 visits. **(A) and (B)** Variation in gene expression levels of VPAC1 (panel A) and VPAC2 (panel B) receptors. Statistical significance was established using the Trend test and non-parametric posteriori test (Dunnett) for intergroup comparison.
(TIF)

S2 Fig VPAC1 protein expression studied by Western Blot analysis of PBMCs lysates from patients with RA with differential DAS28 levels. **A)** Samples from a couple of patients in visit 1 with low and high DAS28 level, respectively. A representative example of two analyses with similar results is shown. **B)** Samples from one patient in two consecutive visits during the follow-up, after and before receiving treatment. In both panels protein bands were densitometrically analyzed and normalized against beta-actin intensity. Pictures are a representative example. **ARC**: Arthritis Register Code for each patient. **V**: Visit number.The protocol of PEARL study does not include storing samples for intracellular proteins analysis. Therefore, it was not possible to perform the protein analysis during the course of the disease in parallel to gene expression studies and it was only conducted on a few patient samples.
(TIF)

S3 Fig Variations in the relative proportions of different cell populations of PBMCs related to the administration of combined therapy. **A)** Proportion of lymphocytes in patients with no treatment or with combined therapy. **B)** Proportion of monocytes in patients with no treatment or with combined therapy. **C)** Representation of the ratio lymphocytes/monocytes in patients with no treatment or with combined therapy. Statistical significance was establish by means of a Kruskal-Wallis test: p<0,05.
(TIF)

S1 Table Ct Values of real-time PCR assay. Data are shown as the mean ± standard deviation.
(DOC)

S2 Table Association between VPAC1 and VPAC2 expression and cell population proportions in patients with combined therapy. The association was studied by means of a univariate GEE (generalized estimating equations method).
(DOC)
