# Supplementary material for: Clinical Relevance of VPAC1 Receptor Expression in Early Arthritis: Association with IL-6 and Disease Activity
Source: PLoS One. 2016 Feb 16;11(2):e0149141. doi: 10.1371/journal.pone.0149141 (PMC4755558; doi:10.1371/journal.pone.0149141)
Supplement: S1 Appendix — (DOC) [file pone.0149141.s001.doc]

**S1 Appendix. Materials and Methods of Western blot analysis of VPAC1 receptor.**

Protein extracts from PBMCs samples of RA patients were obtained in ice-cold radioimmunoprecipitation assay buffer (50mM Tris-HCl pH 7.5, 150mM NaCl, 30mM NaF, 5mM EDTA, 1% Triton X-100, 1% NP-40, 0.1% SDS, 1mM DTT, 1mM sodium orthovanadate, protease inhibitor cocktail). 10 μg of VPAC1 protein extract was subjected to 10% SDS-PAGE and transferred to PVDF membrane (BioRad). After blocking, membrane was incubated overnight at 4ºC with rabbit polyclonal anti-human VPAC1 (1:12,000; ThermoScientific). Mouse anti β-actin (1:15,000, Sigma-Aldrich, St. Louis, USA), was used as a loading control. Horseradish peroxidase conjugated secondary antibody (1:10,000, Santa Cruz Biotechnology, Santa Cruz, USA) was used. Proteins were detected using Western Blotting Luminol reagent (Santa Cruz Biotechnology, Santa Cruz, USA), analyzed using the Bio-Rad Quantity One program and normalized against β-actin.
