# Supplementary figures and images for: Clinical Relevance of VPAC1 Receptor Expression in Early Arthritis: Association with IL-6 and Disease Activity
Source: PLoS One. 2016 Feb 16;11(2):e0149141. doi: 10.1371/journal.pone.0149141 (PMC4755558; doi:10.1371/journal.pone.0149141)

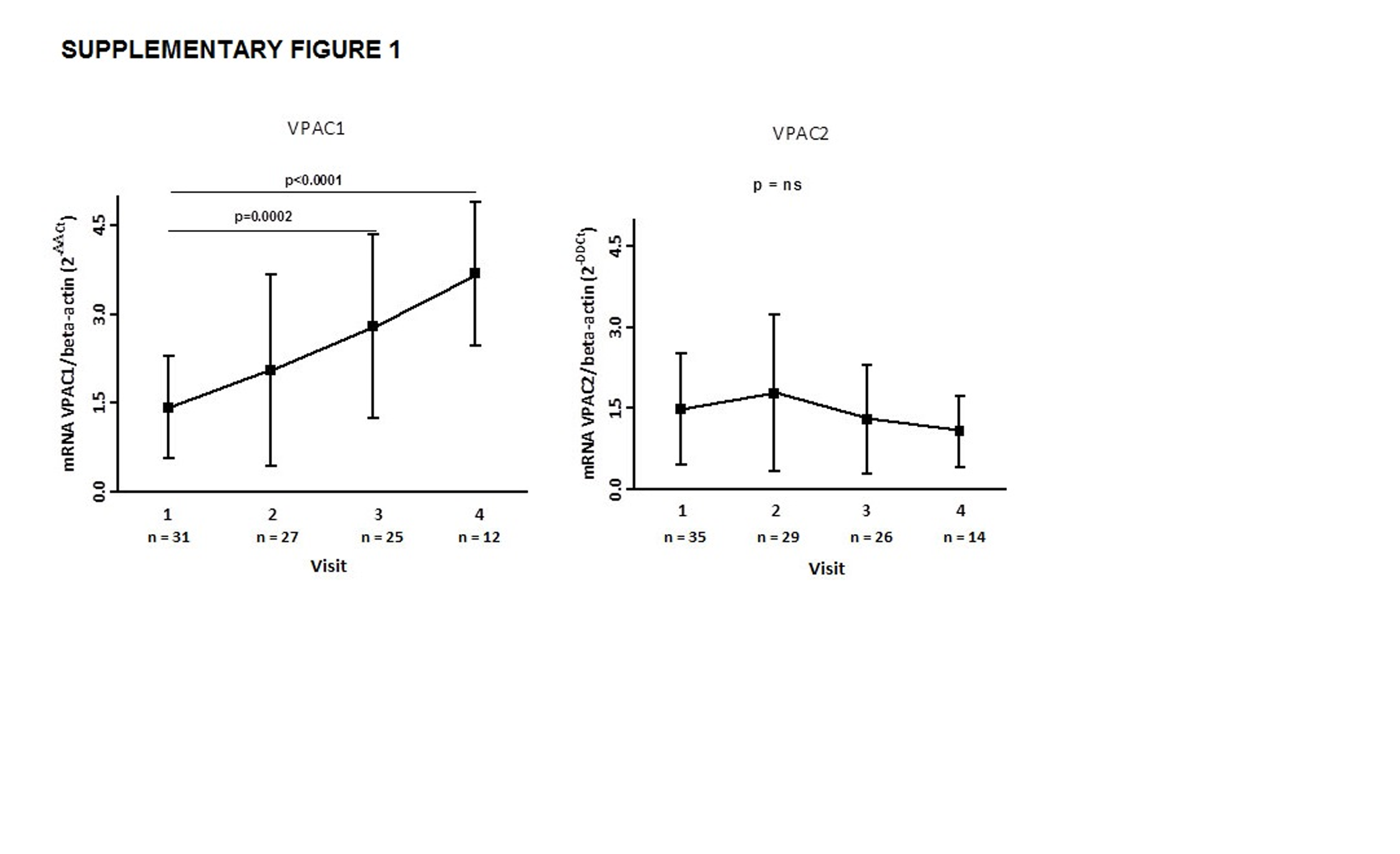

Supplement: S1 Fig — (A) and (B) Variation in gene expression levels of VPAC1 (panel A) and VPAC2 (panel B) receptors. Statistical significance was established using the Trend test and non-parametric posteriori test (Dunnett) for intergroup comparison. (TIF) [file pone.0149141.s002.TIF]

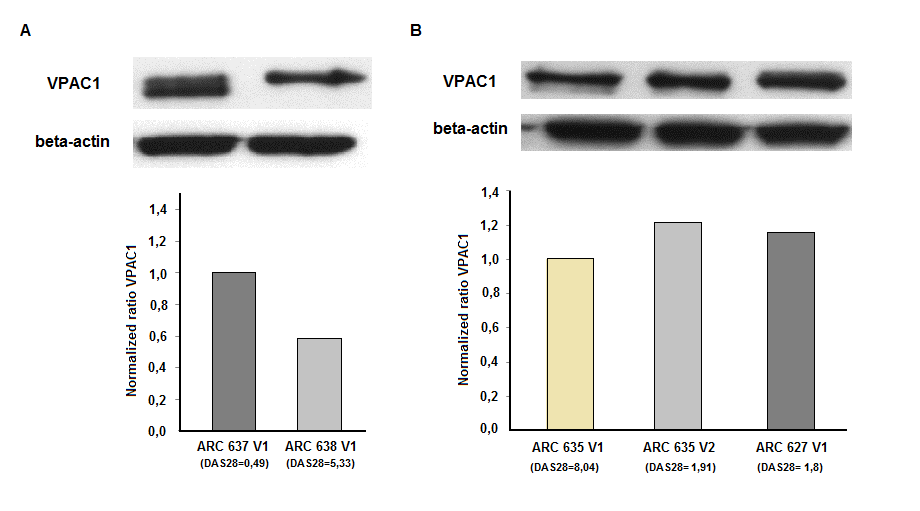

Supplement: S2 Fig — A) Samples from a couple of patients in visit 1 with low and high DAS28 level, respectively. A representative example of two analyses with similar results is shown. B) Samples from one patient in two consecutive visits during the follow-up, after and before receiving treatment. In both panels protein bands were densitometrically analyzed and normalized against beta-actin intensity. Pictures are a representative example. ARC: Arthritis Register Code for each patient. V: Visit number.The protocol of PEARL study does not include storing samples for intracellular proteins analysis. Therefore, it was not possible to perform the protein analysis during the course of the disease in parallel to gene expression studies and it was only conducted on a few patient samples. (TIF) [file pone.0149141.s003.TIF]

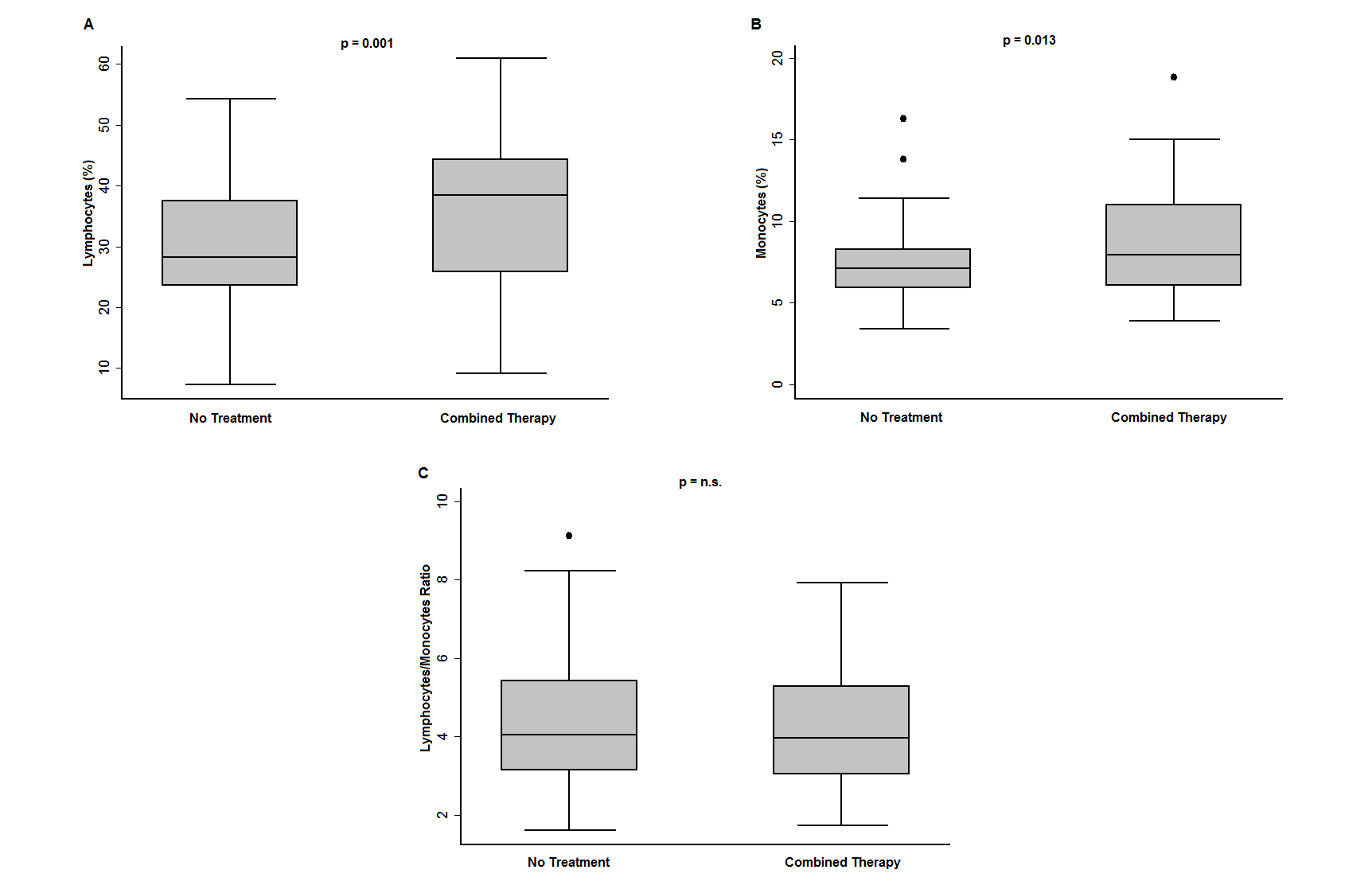

Supplement: S3 Fig — A) Proportion of lymphocytes in patients with no treatment or with combined therapy. B) Proportion of monocytes in patients with no treatment or with combined therapy. C) Representation of the ratio lymphocytes/monocytes in patients with no treatment or with combined therapy. Statistical significance was establish by means of a Kruskal-Wallis test: p<0,05. (TIF) [file pone.0149141.s004.TIF]
