# Supplementary material for: Clinical Relevance of VPAC1 Receptor Expression in Early Arthritis: Association with IL-6 and Disease Activity
Source: PLoS One. 2016 Feb 16;11(2):e0149141. doi: 10.1371/journal.pone.0149141 (PMC4755558; doi:10.1371/journal.pone.0149141)
Supplement: S1 Table — Data are shown as the mean ± standard deviation. (DOC) [file pone.0149141.s005.doc]

**S1 Table.** Ct Values of real-time PCR assay.

|  | **Visits** | | | | |
| --- | --- | --- | --- | --- | --- |
| **Gene** | **V1** | **V2** | **V3** | **V4** | **V5** |
| β−Actin | 19.6±1.6 | 19.7±1.0 | 19.4±1.4 | 19.2±1.2 | 19.5±1.4 |
| VPAC1 | 27.8±1.8 | 27.1±1.7 | 26.3±2.0 | 25.6±1.7 | 26.5±1.4 |
| VPAC2 | 29.3±1.8 | 29.0±1.3 | 29.3±1.8 | 29.5±1.5 | 30.2±1.6 |
| IL-6 | 30.5±1.5 | 31.3±1.5 | 32.3±1.3 | 31.8±1.6 | 32.4±1.4 |

Data are shown as the mean ± standard deviation.
