# Supplementary material for: Clinical Relevance of VPAC1 Receptor Expression in Early Arthritis: Association with IL-6 and Disease Activity
Source: PLoS One. 2016 Feb 16;11(2):e0149141. doi: 10.1371/journal.pone.0149141 (PMC4755558; doi:10.1371/journal.pone.0149141)
Supplement: S2 Table — The association was studied by means of a univariate GEE (generalized estimating equations method). (DOC) [file pone.0149141.s006.doc]

|  | **VPAC1** | | **VPAC2** | |
| --- | --- | --- | --- | --- |
|  | **Β Coeff.** | **p-value** | **Β Coeff.** | **p-value** |
| **Lymphocytes (%)** | 0.164 | 0.020 | 0.039 | 0.310 |
| **Monocytes (%)** | 0.821 | 0.000 | 0.066 | 0.633 |
| **Lymphocytes/Monocytes Ratio** | -0.596 | 0.469 | 0.397 | 0.254 |

**S2 Table.** Association between VPAC1 and VPAC2 expression and cell population proportions in patients with combined therapy.

The association was studied by means of a univariate GEE (generalized estimating equations method).
